# Development and validation of a nomogram for predicting the incidence of infectious events in patients with idiopathic inflammatory myopathies

**DOI:** 10.3389/fimmu.2025.1471152

**Published:** 2025-03-05

**Authors:** Luwei Yang, Guihua Fan, Lijuan Zhang, Binbin Zhou, Xiaomin Dai, Zongfei Ji, Lingying Ma, Zhuojun Zhang, Huiyong Chen, Qiang Yu, Lili Ma, Lindi Jiang, Ying Sun

**Affiliations:** ^1^ Department of Rheumatology, Zhongshan Hospital (Xiamen), Fudan University, Xiamen, China; ^2^ Department of Rheumatology, Zhongshan Hospital, Fudan University, Shanghai, China

**Keywords:** idiopathic inflammatory myopathies, severe infection, nomogram, risk prediction, Kaplan-Meier analysis

## Abstract

**Background:**

Infection is a leading cause of mortality in idiopathic inflammatory myopathies (IIMs). This study aimed to develop a nomogram for predicting severe infection risk in IIM patients.

**Methods:**

Patients with IIMs admitted to Zhongshan Hospital, Fudan University, from January 2015 to January 2022 were enrolled. They were randomly divided into derivation (70%) and validation (30%) sets. Univariate and multivariate Cox regression identified independent risk factors for severe infection, and the Akaike information criterion (AIC) was applied for model selection. A nomogram was constructed to predict severe infection risks at 6 months, 1 year, and 3 years. Predictive accuracy and discriminative ability were evaluated using the concordance index (C-index), calibration curves, and the area under the receiver operating characteristic curve (AUC). Decision curve analysis (DCA) assessed clinical utility. Kaplan-Meier (K-M) curves were used to analyze survival differences between high- and low-risk groups stratified by nomogram scores.

**Results:**

Among 263 IIM patients, 81 experienced 106 severe infection events, with lower respiratory tract infections being the most common (47.2%). Independent risk factors included age at onset (HR 1.024, 95% CI 1.002-1.046, *p*=0.036), lactate dehydrogenase (HR 1.002, 95% CI 0.999-1.005, *p*=0.078), HRCT score (HR 1.004, 95% CI 1.001-1.006, *p*=0.002), and lymphocyte count (HR 0.48, 95% CI 0.23-0.99, *p*=0.048). The nomogram demonstrated strong predictive performance, with AUCs of 0.84, 0.83, and 0.78 for 6 months, 1 year, and 3 years in the derivation set, and 0.91, 0.77, and 0.64 in the validation set. Calibration curves showed good agreement between predicted and observed risks, while DCA demonstrated significant net benefit over individual predictors. Kaplan-Meier curves revealed significant differences in the cumulative risk of severe infection between high- and low-risk groups. Further validation in DM and ASS subgroups demonstrated that the nomogram effectively predicted severe infections, with AUCs of 0.86, 0.81, and 0.73 for DM and 0.86, 0.83, and 0.74 for ASS at 6 months, 1 year, and 3 years, respectively.

**Conclusion:**

We have developed a new nomogram to predict severe infection risk in IIM patients at 6 months, 1 year, and 3 years. This model aids clinicians and patients in formulating treatment and follow-up strategies.

## 1 Introduction

Idiopathic inflammatory myopathies (IIMs) are a heterogeneous group of systemic autoimmune diseases, including dermatomyositis (DM), polymyositis (PM), antisynthetase syndrome (ASS), and immune-mediated necrotizing myopathy (IMNM). These conditions are characterized by immune-mediated damage to skeletal muscles and other visceral organs (1, 2). With the increasing understanding of these diseases and advancements in diagnostic techniques, the incidence of IIMs has been steadily rising, estimated at 0.2 to 2 cases per 100,000 person-years (3). However, despite improvements in diagnostic and therapeutic approaches, the prognosis remains poor, with the overall mortality risk for IIM patients being 3.7 times higher than that of the general population (95% CI 3.2–4.4) (4). Specifically, the 10-year mortality rate for patients with DM can be as high as 42% to 74% (5). Infections are the leading cause of severe complications and mortality in patients with IIM. Reports indicate that up to 26% of these patients experience infectious complications, including skin, respiratory, and urinary tract infections (6). The risk of death increases by 4.2-fold following an infection, making it the strongest predictor of mortality in IIM patients (7). Therefore, early identification and intervention for high-risk patients are crucial.

While previous studies have explored the risk factors for infection in IIM patients, a study from France indicated that patients with dysphagia, interstitial lung disease (ILD), myalgia, concurrent malignancies, and those treated with methotrexate are more susceptible to infections, with the highest risk observed in ILD patients (8). Ge YP et al. identified, through multivariate analysis, the risk factors for infection in IIM patients as high-dose glucocorticoid pulse therapy, lymphopenia, ILD, anti- anti-melanoma differentiation-associated protein 5 (MDA5) antibody positivity, and age >50 years (9). However, these assessments often consider the independent effect of each variable, potentially overlooking the complex interactions and relationships between different factors.

In recent years, nomogram risk prediction models have been widely used for prognostic evaluation in various diseases. These models integrate clinical characteristics and laboratory results to provide a user-friendly graphical interface, facilitating clinical use and aiding in clinical decision-making. However, no predictive models specifically for infection risk in IIM patients have been published. This study aims to fill this gap by systematically exploring the risk factors for severe infection in IIM patients and developing a nomogram that incorporates these factors to help clinicians identify high-risk IIM patients and make informed clinical decisions.

## 2 Materials and methods

### 2.1 Study population

**Figure 1:**
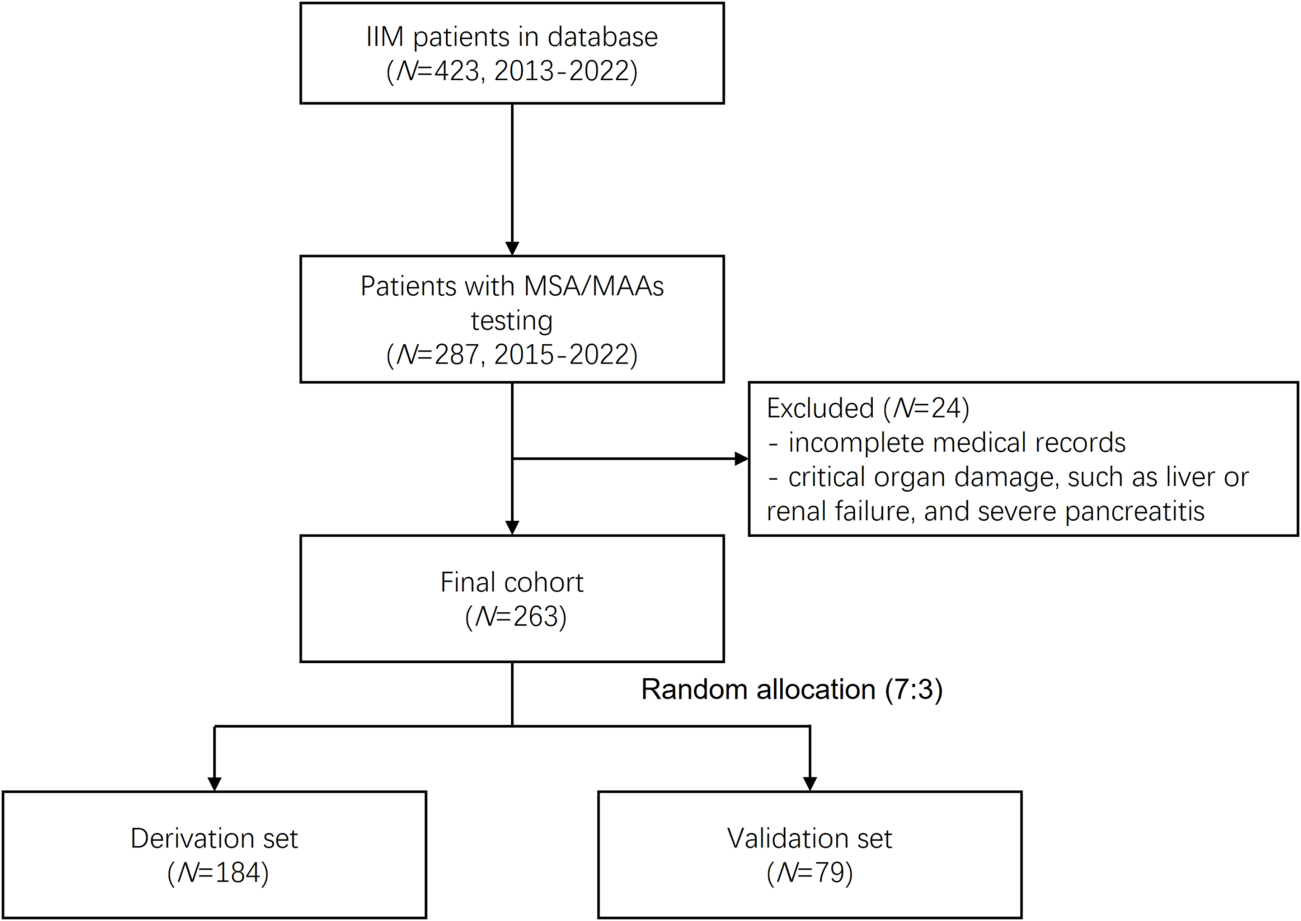
Flow-chart for patient selection process.

This study retrospectively analyzed 423 patients with IIMs registered in the Zhongshan Hospital myositis database between January 2013 and January 2022. From this cohort, 287 patients who underwent myositis-specific antibodies (MSAs) and myositis-associated antibodies (MAAs) testing between January 2015 to January 2022 were preliminarily selected. After excluding cases with incomplete medical records and those with critical organ damage (including liver failure, renal failure, and severe pancreatitis), 263 patients were ultimately enrolled. These patients were randomly allocated into derivation (n=184) and validation (n=79) sets in a 7:3 ratio. The complete selection process is illustrated in **Figure 1**. All diagnoses adhered to established criteria: the 1975 Bohan & Peter criteria for PM and DM, the European Neuromuscular Centre (ENMC) guidelines for IMNM (10), and the 2010 Connors criteria for ASS (11). ILD was confirmed when the following conditions were satisfied (12): the core criterion was that high resolution computer tomography (HRCT) demonstrated typical interstitial findings, including ground-glass opacities, alveolar consolidation, thickening of the interlobular septum, subpleural lines, reticular shadows with cavity formation or honeycombing, and traction bronchiectasis. Supportive evidence included the presence of respiratory symptoms (such as dry cough, wheezing, and exertional dyspnea), clinical signs (including Velcro rales at lung bases and digital clubbing), and pulmonary function tests revealing restrictive defects (defined as total lung capacity [TLC] and diffusing capacity of the lungs for carbon monoxide [DLCO] values less than 80% of the predicted values). Additionally, secondary causes of ILD, including drug-related interstitial changes and other potential etiologies (such as heart failure), were excluded. The study received approval from the ethics committee of Zhongshan Hospital, Fudan University [B2013-115 (3)], and written informed consent was obtained from all participants.

### 2.2 Clinical data collection

Medical records were extracted from the hospital’s electronic records system, encompassing patient history, laboratory test results, and imaging findings. All extracted data were collected at the time of the patients’ initial visit. The risk factors for infectious events assessed included age, sex, current and past smoking status, semi-quantitative evaluation of ILD via a standardized thoracic HRCT scoring system, total lymphocyte, and lymphocyte subset counts, initial dosage of corticosteroids at the onset of treatment, and the specific types of immunosuppressants administered. The HRCT score was ascertained using validated radiological criteria, while deviations in lymphocyte counts and subpopulations were determined based on the reference ranges provided by the local laboratory services. Laboratory tests performed included complete blood counts, lymphocyte subset analysis, biochemical assays, and microbiological cultures when indicated, all conducted at baseline and prior to the commencement of therapy. The diagnosis of severe infectious events was grounded on clinical manifestations, laboratory confirmation, and pathogen detection, conclusively verified by infectious disease specialists.

### 2.3 Thoracic HRCT score

The classic six-zone method was employed to partition the lung anatomy into six regions (13): upper left, upper right, middle left, middle right, lower left, and lower right. The boundaries were defined with the carina as the upper limit and the lower extent of the pulmonary veins as the inferior boundary, with the remaining lung fields constituting the middle zone. Changes in lung structure and corresponding scores were categorized as follows: (1) attenuation of normal signal scored 1 point; (2) ground-glass opacities without bronchiectasis scored 2 points; (3) consolidation without bronchiectasis scored 3 points; (4) ground-glass opacities with bronchiectasis scored 4 points; (5) consolidation with bronchiectasis scored 5 points; (6) honeycombing scored 6 points. Each of the six regions was assessed independently. The extent of abnormal imaging findings within each lung zone was estimated in 5% increments, which was then multiplied by the respective scores. The final HRCT score for each patient was determined by averaging the scores of all six regions.

### 2.4 Definition of severe infection

In this study, a severe infection is defined as an infectious event meeting any of the following criteria: (1) Clinical manifestations: The patient exhibits severe symptoms, including, but not limited to, persistent high fever (body temperature ≥38.5°C), difficulty breathing, or hemodynamic instability. (2) Treatment requirements: The condition necessitates intensified therapeutic measures, such as intravenous antibiotics or supportive care. (3) Adjustment of immunosuppressants: Due to the severity of the infection, a modification in the dosage of immunosuppressants is required, including discontinuation or significant reduction, to enhance the body’s resistance to infection. (4) Physician’s decision: Based on a comprehensive evaluation of the patient’s condition and response to treatment, including the presence of clinical symptoms (fever, organ dysfunction, etc) and the need for antiviral therapy or adjustment of immunosuppressive treatment in specific infections like CMV reactivation, the physician decides to implement the above measures.

### 2.5 Outcomes and follow-up procedures

Follow-up was primarily conducted through regular outpatient visits and telephone interviews to ensure comprehensive monitoring. During these visits, patients were evaluated for various health parameters and adherence to treatment protocols. The primary endpoint of the study was the incidence of severe infections within six months. Secondary endpoints included the incidence of severe infections at one and three years, with long-term follow-up outcomes over five years also being closely monitored. While all severe infection events in patients were recorded, only the first severe infection event for each patient was included in the final statistical analysis to avoid double-counting and to ensure data consistency and accuracy, which was used for clinical prediction of severe infections. Data were systematically collected and recorded to facilitate detailed analysis and enhance the accuracy of the study’s findings.

### 2.6 Statistical analysis

To ensure the robustness and discriminative power of the model, patients were randomly divided into derivation and validation sets in a 7:3 ratio using R software. Baseline characteristics were compared between the derivation and validation sets. Continuous variables that were normally distributed were expressed as mean ± standard deviation and compared using the t-test. Continuous variables with a non-normal distribution were summarized as median (interquartile range, IQR) and compared using the Mann-Whitney U test. Categorical variables were presented as counts (proportion) and compared using the Chi-square test or Fisher’s exact test, as appropriate. The overall predictive model for infection events was developed in the derivation set using a multivariable Cox regression model. During the variable selection phase, univariable Cox regression analysis was first conducted, and variables with a *p*-value < 0.05 were considered candidate variables. Spearman’s rank correlation coefficient was then employed to assess the correlation between candidate variables. Subsequently, Cox regression analysis was performed on all subsets based on the Akaike Information Criterion (AIC), and the model with the lowest AIC value was selected as the predictive model. The proportional hazards assumption of the model was evaluated using Schoenfeld residuals. The infection risk score was calculated by summing the risk points corresponding to each weighted covariate. Individuals were then categorized into different levels of infection risk based on their risk scores and the optimal cutoff value determined by ROC curve analysis. The predictive model was assessed for discrimination and calibration in both the derivation and validation sets. Receiver Operating Characteristic (ROC) curves were plotted, and the area under the curve (AUC) was measured. Model performance was further evaluated by analyzing the separation between the ROC curves. Calibration plots were constructed by comparing the actual rates of infection events with the probabilities predicted by the model. Additionally, clinical decision curves (DCA) were drawn to evaluate the net benefit of the predictive model across different thresholds by balancing the relative harms of false-positive and false-negative diagnostic results. All analyses were performed using R software version 4.3.1.

## 3 Results

### 3.1 Clinical characteristics and features

Between 2015 and 2022, a total of 263 patients diagnosed with IIMs were treated at Zhongshan Hospital, Fudan University. These patients were divided into derivation and validation sets in a 7:3 ratio, with 184 patients in the derivation set and 79 patients in the validation set. We thoroughly recorded the patients’ baseline clinical symptoms, physical signs, laboratory tests, HRCT scores, and corticosteroid dosage. Based on the specific types of MSAs, we categorized the patients into four groups: anti-MDA5 antibody, anti-synthetase antibody, other myositis antibodies excluding anti-MDA5 and anti-synthetase, and negative myositis antibodies. Additionally, based on the characteristics of immunosuppressants, we classified them into two categories: Class I (cyclophosphamide, mycophenolate mofetil, cyclosporine, tacrolimus, azathioprine, tofacitinib) and Class II (methotrexate, leflunomide, Tripterygium wilfordii Hook F, hydroxychloroquine, paeoniflorin, etc.). There were no significant differences in baseline characteristics between the two groups in these aspects, as detailed in **Table 1**.

**Table 1:** Baseline characteristics of the derivation set and validation set.

	Derivation Set	Validation Set	*p-*Value
No. of patients	184	79	–
Female, No. (%)	110 (59.8)	56 (70.9)	0.116
Age, median (IQR), ys	57.0 (48.0,66.0)	57.0 (44.0,67.0)	0.761
Disease duration, median (IQR), ms	3.0 (1.0,16.0)	4.0 (1.0,15.0)	0.667
Follow-up duration, median (IQR), ms	26.0 (17.0,41.0)	22.0 (14.0;38.0)	0.093
Diagnosis, No. (%)			0.685
DM	92 (50.0)	34 (43.0)	
PM	18 (9.8)	7 (8.9)	
ASS	64 (34.8)	33 (41.8)	
IMNM	10 (5.4)	5 (6.3)	
Clinical signs			
Muscle weakness, No. (%)	99 (53.8)	45 (57.0)	0.736
Arthritis, No. (%)	25 (13.6)	14 (17.7)	0.499
Mechanics hand, No. (%)	71 (38.6)	28 (35.4)	0.731
Heliotrope rash, No. (%)	84 (45.7)	36 (45.6)	1.000
Gottron’s sign, No. (%)	99 (53.8)	37 (46.8)	0.367
ILD, No. (%)	114 (62.0)	46 (58.2)	0.667
HRCT score, mean (SD)	18.0 (0.0,79.2)	18.0 (0.0,62.0)	0.730
Laboratory results			
WBC, median (IQR),×10^9^/L	7.8 (5.7,10.3)	7.5 (5.9,9.2)	0.561
NEUT, median (IQR),×10^9^/L	5.6 (3.7,8.5)	5.3 (4.0,7.3)	0.430
LYM, median (IQR),×10^9^/L	1.0 (0.7,1.3)	1.1 (0.8,1.6)	0.176
Alb, mean (SD), g/L	35.8 (4.9)	35.8 (4.7)	0.966
ALT, median (IQR), U/L	38.0 (22.0,64.2)	34.0 (18.5,55.0)	0.214
AST, median (IQR), U/L	38.0 (22.5,75.5)	36.0 (21.0,75.2)	0.901
LDH, median (IQR), U/L	314 (239,414)	302 (230,456)	0.958
CK, median (IQR), U/L	324 (109,1494)	531 (167,1898)	0.247
CRP, median (IQR), mg/L	5.3 (2.5,9.8)	4.5 (2.6,10.2)	0.846
IgG, median (IQR), g/L	15.1 (11.3,19.5)	13.5 (10.8,19.0)	0.497
IgA, median (IQR), g/L	2.40 (1.70,3.30)	2.30 (1.70,3.05)	0.970
IgM, median (IQR), g/L	1.70 (1.08,2.50)	1.70 (1.05,2.20)	0.536
Myositis-specific autoantibodies, No. (%)			0.330
Negative	37 (20.1)	16 (20.3)	
Anti-MDA5	32 (17.4)	6 (7.6)	
Anti-synthetase	64 (34.8)	33 (41.8)	
Anti-SRP	12 (6.5)	5 (6.3)	
Others	39 (21.2)	19 (24.1)	
Prednisone dose, median (IQR), mg/day	40.0 (30.0,50.0)	40.0 (40.0,50.0)	0.642
Immunosuppressor, No. (%)			1.000
Class I	99 (53.8)	43 (54.4)	
Class II	85 (46.2)	36 (45.6)	

* IQR, Interquartile Range; SD, standard deviation; DM, Dermatomyositis; PM, Polymyositis; ASS, Anti-synthetase Syndrome; IMNM, Immune-mediated Necrotizing Myopathy; ILD, Interstitial Lung Disease; WBC, White Blood Cell; NEUT, Neutrophil; LYM, Lymphocyte; Alb, Albumin; ALT, Alanine Aminotransferase; AST, Aspartate Aminotransferase; LDH, Lactate Dehydrogenase; CRP, C-Reactive Protein; MDA5, Melanoma Differentiation-Associated protein 5; SRP, Signal Recognition Particle; CK, Creatine Kinase.

** All laboratory results presented in this table are baseline measurements taken at the time of initial diagnosis and prior to the commencement of therapy.

### 3.2 Severe infection events

During the observation period, a total of 106 severe infection events were observed among 263 patients. Among those infected, the majority (65 patients, 80.2%) experienced only one severe infection event, while the remainder (16 patients, 19.8%) experienced recurrent (≥2 times, up to 5 times) severe infections. The median time from the diagnosis of IIMs to the first severe infection event was 4.0 (1.0,15.0) months. Out of the patients with severe infections, 42 succumbed to death. Compared to patients who did not experience severe infections during the observation period, those who did had longer follow-up durations. The most common severe infections were lower respiratory tract infections (47.2%), urinary system infections (17.9%), and gastrointestinal infections (15.1%), with 6.6% of the patients experiencing bloodstream infections (**Table 2**). Bacterial infections (26.4%) were the most common pathogens identified, with Escherichia coli being the most prevalent at 11 cases (10.4%). This was followed by cytomegalovirus with 9 cases (8.5%), Klebsiella pneumonia with 6 cases (5.7%), and Acinetobacter baumannii with 5 cases (4.7%). However, a significant proportion of the infections (39.6%) had no identified pathogen (**Table 2**).

**Table 2:** Severe infection assessment of the derivation set and validation set.

	Total	Derivation Set	Validation Set
No. of patients	263	184	79
Infected Patients	81	57	24
Severe Infection Events	106	78	28
Death Events, No. (%)	42 (16.0)	28 (15.2)	14 (17.7)
Time of First Infection Appearance, ms	4.0 (1.0,15.0)	3.0 (1.0,15.2)	4.0 (1.0,14.0)
Frequency of Severe Infections			
*1 time*	65	44	21
*2-4 times*	15	12	3
*≥5 times*	1	1	0
Infection Type			
*Lower Respiratory Tract Infection, No. (%)*	50 (47.2)	38 (48.7)	12 (42.9)
*Urinary Tract Infection, No. (%)*	19 (17.9)	15 (19.2)	4 (16.7)
*Gastrointestinal Infection, No. (%)*	16 (15.1)	12 (15.3)	4 (16.7)
*Bloodstream Infection, No. (%)*	7 (6.6)	4 (5.1)	3 (12.5)
*Others, No. (%)*	14 (13.2)	9 (11.5)	5 (20.8)
Pathogen Type			
Bacteria, No. (%)	28 (26.4)	21 (26.9)	7 (25.0)
*Escherichia coli*	11 (10.4)	8 (10.3)	3 (10.7)
*Klebsiella pneumoniae*	6 (5.7)	4 (5.1)	2 (7.1)
*Acinetobacter baumannii*	5 (4.7)	3 (3.8)	2 (7.1)
*Other*	6 (5.7)	5 (6.4)	1 (3.6)
Fungi, No. (%)	7 (6.6)	4 (5.1)	3 (10.7)
*Moniliasis*	3 (2.8)	2 (2.6)	1 (3.6)
*Aspergillus*	3 (2.8)	1 (1.3)	1 (3.6)
*Cryptococcus*	1 (1.0)	0 (0)	1 (3.6)
Virus, No. (%)	13 (12.3)	9 (11.5)	4 (14.3)
*CMV*	9 (8.5)	6 (7.7)	3 (10.7)
*Other*	4 (3.8)	3 (3.8)	1 (3.6)
Mixed infection, No. (%)	16 (15.1)	11 (14.1)	5 (17.9)
*PJP+Other*	6 (5.7)	4 (5.1)	2 (7.1)
*Non-PJP Mixed Infection*	10 (9.4)	7 (9.0)	3 (10.7)
Unknown, No. (%)	42 (39.6)	33 (42.3)	9 (32.1)

* CMV, Cytomegalovirus; PJP, Pneumocystis Jiroveci Pneumonia.

### 3.3 Nomogram prediction of first severe infection

The study considered multiple continuous and categorical variables as potential predictors. The continuous variables included patient age, disease duration, CRP, NEUT, LYM, LDH, HRCT score, and initial prednisone dosage. The categorical variables included the presence of arthritis, rash, MSA types, and the type of immunosuppressant used. These variables were selected as candidate predictors based on univariate Cox regression analysis.

**Figure 2:**
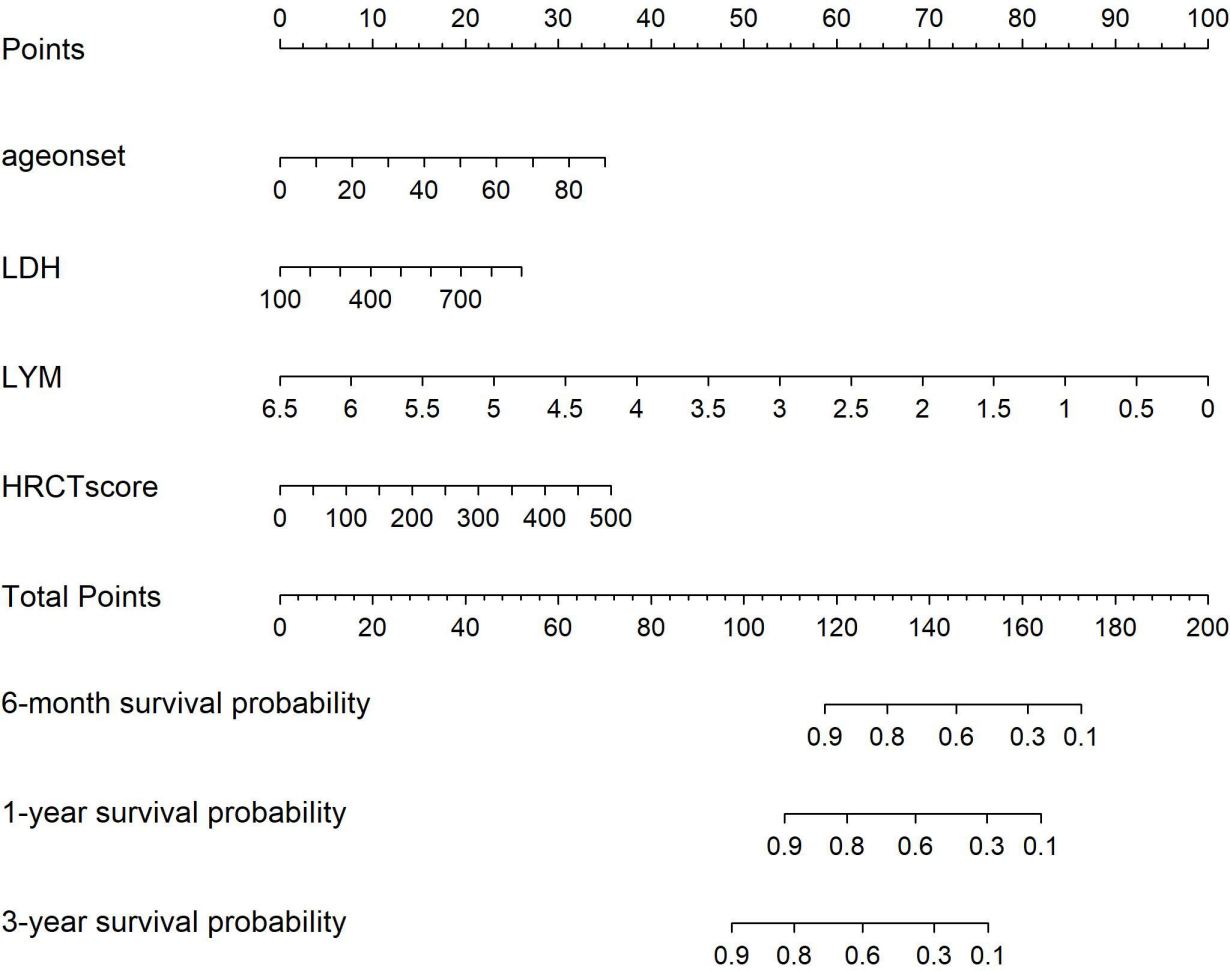
Nomogram for Severe infection prediction (HALL model). Draw a perpendicular line from each risk factor’s corresponding axis up to the line labeled ‘Points’. Sum the points for all risk factors, then draw a line down from the ‘Total Points’ axis until it intersects each risk axis to determine the 6-month, 1-year, and 3-year probabilities of severe infection.

After conducting Cox regression analysis on all subsets, the model with the lowest AIC value was chosen as the final prediction model. This model incorporated four predictive indicators: HRCT score, Age, LYM, and LDH (HALL model, **Table 3**). The nomogram designed to predict the risk of first severe infections at 6 months, 1 year, and 3 years is shown in **Figure 2**.

**Table 3:** The prediction model based on multivariable Cox regression.

Variables	Univariate analysis		Multivariate analysis		Final analysis	
	Hazard Ratio (95%CI)	*p-*value	Hazard Ratio (95%CI)	*p-*value	Hazard Ratio (95%CI)	*p-*value
Age	1.03 (1.01-1.05)	0.003	1.024 (1.002-1.046)	0.036	1.0220 (1.0004-1.0440)	0.046
Sex						
female	Reference					
male	1.31 (0.77-2.21)	0.319				
Disease duration	0.997 (0.989-1.005)	0.497				
Diagnosis						
DM	Reference					
PM	0.28 (0.07-1.16)	0.079				
ASS	0.90 (0.52-1.58)	0.718				
IMNM	0.41 (0.10-1.73)	0.226				
Muscle weakness						
No	Reference					
Yes	0.91 (0.54-1.53)	0.495				
Arthritis						
No	Reference					
Yes	0.76 (0.34-1.68)	0.657				
Heliotrope rash						
No	Reference					
Yes	1.28 (0.76-2.15)	0.361				
Gottron sign						
No	Reference					
Yes	1.30 (0.76-2.21)	0.340				
Mechanical hand						
No	Reference					
Yes	1.18 (0.69-2.02)	0.538				
ILD						
No	Reference					
Yes	1.83 (1.03-3.27)	0.041	0.92 (0.37-2.29)	0.859		
MSA						
Negative	Reference					
Anti-MDA5	3.46 (1.34-8.96)	0.011	1.90 (0.64-5.60)	0.245		
Anti-synthetase	2.05 (0.82-5.12)	0.123	1.31 (0.49-3.49)	0.589		
Anti-SRP	1.26 (0.31-5.04)	0.749	0.85 (0.19-3.84)	0.836		
Others	1.85 (0.70-4.93)	0.215	1.52 (0.53-4.35)	0.434		
HRCTscore	1.005 (1.004-1.007)	<0.001	1.004 (1.001-1.006)	0.002	1.004 (1.002-1.006)	<0.001
Initial prednisone dosage	1.003 (0.999-1.005)	0.059	1.002 (0.998-1.005)	0381		
Immunosuppressor						
Class I	Reference					
Class II	1.01 (0.60-1.71)	0.956				
WBC	1.02 (0.96-1.09)	0.485				
NEUT	1.06 (0.99-1.13)	0.093				
LYM	0.30 (0.15-0.60)	<0.001	0.48 (0.23-0.99)	0.048	0.42 (0.21-0.87)	0.018
ALT	1.0032 (1.0001-1.0062)	0.040	0.999 (0.995-1.005)	0.975		
AST	1.002 (0.999-1.004)	0.164				
Alb	1.02 (0.96-1.07)	0.590				
LDH	1.002 (1.001-1.003)	0.001	1.002 (0.999-1.005)	0.078	1.0018 (1.0002-1.0034)	0.025
CK	1.00001 (0.99987-1.00008)	0.681				
CRP	1.001 (0.990-1.012)	0.809				

* CI, Confidence Interval; DM, Dermatomyositis; PM, Polymyositis; ASS, Anti-synthetase Syndrome; IMNM, Immune-mediated Necrotizing Myopathy; ILD, Interstitial Lung Disease; WBC, White Blood Cell; NEUT, Neutrophil; LYM, Lymphocyte; Alb, Albumin; ALT, Alanine Aminotransferase; AST, Aspartate Aminotransferase; LDH, Lactate Dehydrogenase; CRP, C-Reactive Protein; MDA5, Melanoma Differentiation-Associated protein 5; SRP, Signal Recognition Particle; CK, Creatine Kinase.

### 3.4 Validation of the nomogram

**Figure 3:**
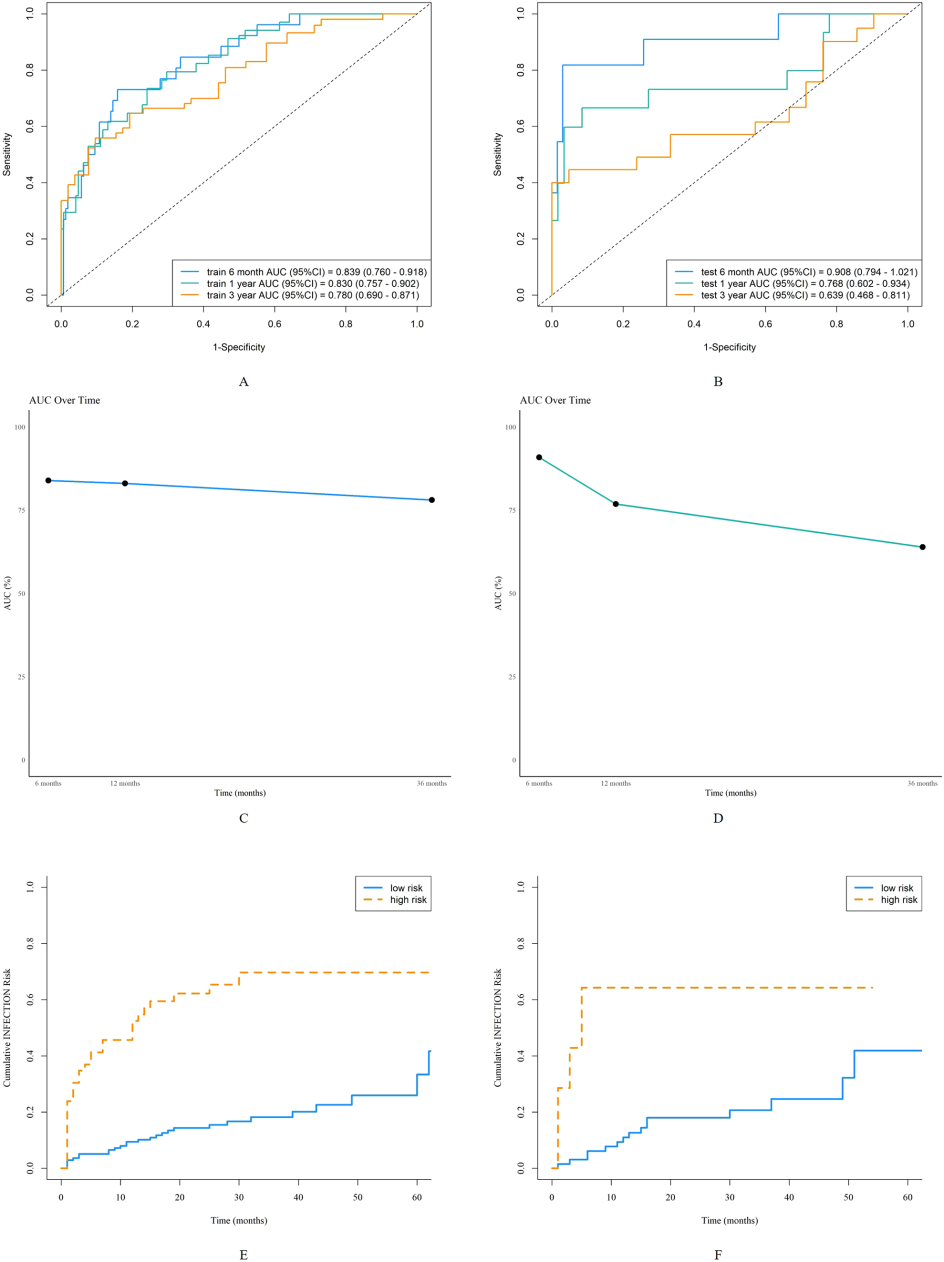
Discrimination of the nomogram in the derivation and validation sets. Receiver operating characteristic curves for 6-month, 1-year, and 3-year severe infection predictions in the derivation set **(A)** and validation set **(B)**. The area under the curve of the nomogram for predicting 6-month to 3-year severe infection in the derivation data **(C)** and validation data **(D)**. Kaplan-Meier curves of the derivation set **(E)** and validation set **(F)** stratified into high-risk and low-risk groups based on the cutoff value of 54.

**Figure 4:**
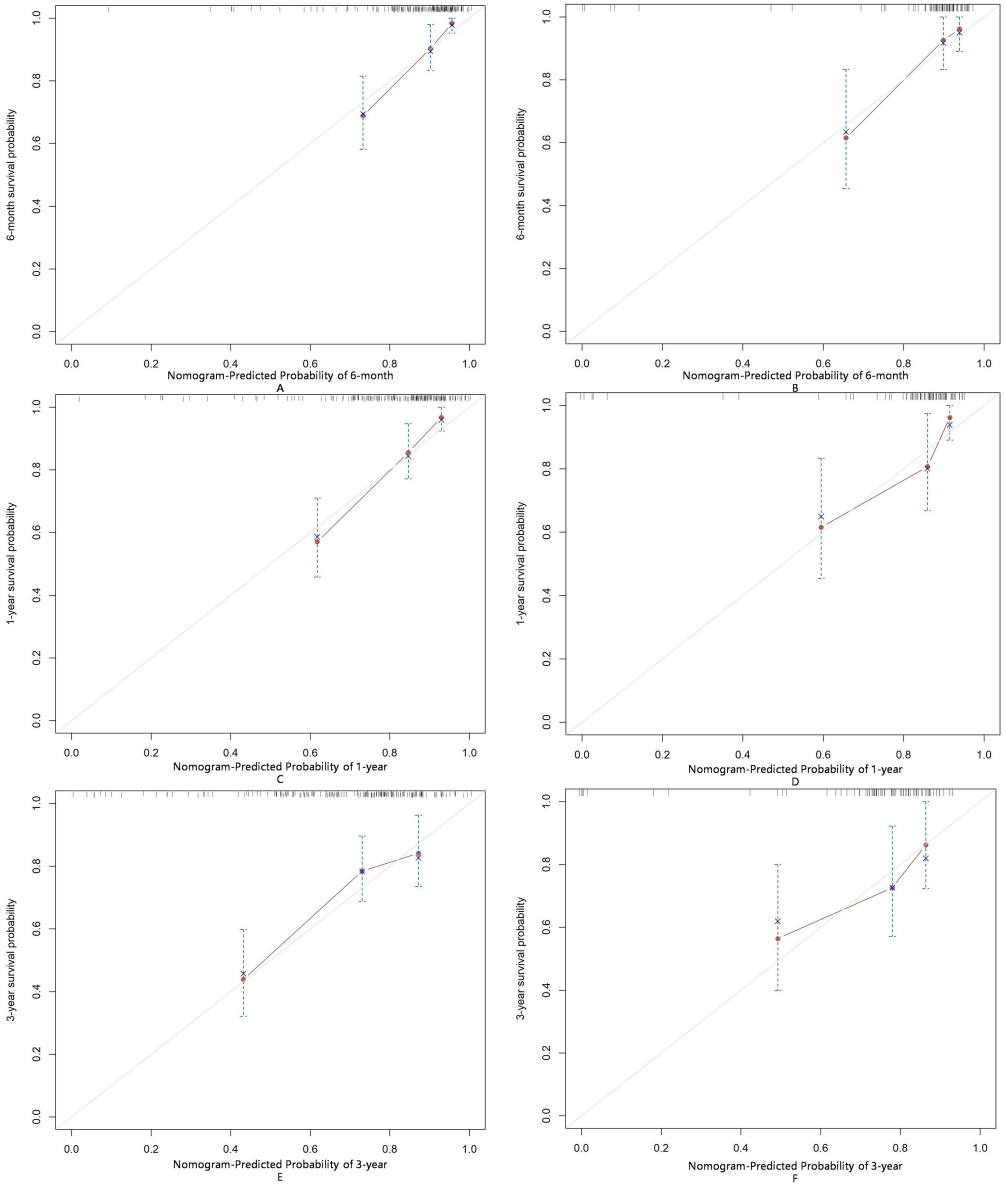
Calibration of the nomogram for infection predictions at 6-month **(A, B)**, 1-year **(C, D)**, and 3-year **(E, F)** intervals in the derivation and validation sets. Data are sourced from the derivation set **(A, C, E)** and the validation set **(B, D, F)**. The nomogram predicted cumulative incidence rates of infection, stratified into equally sized subgroups. For each subgroup, the average predicted infection incidence rate is plotted against the observed infection incidence rate. Vertical lines represent the 95% confidence intervals for the infection incidence rates. The gray lines serve as reference lines, indicating the ideal position for the nomogram.

**Figure 5:**
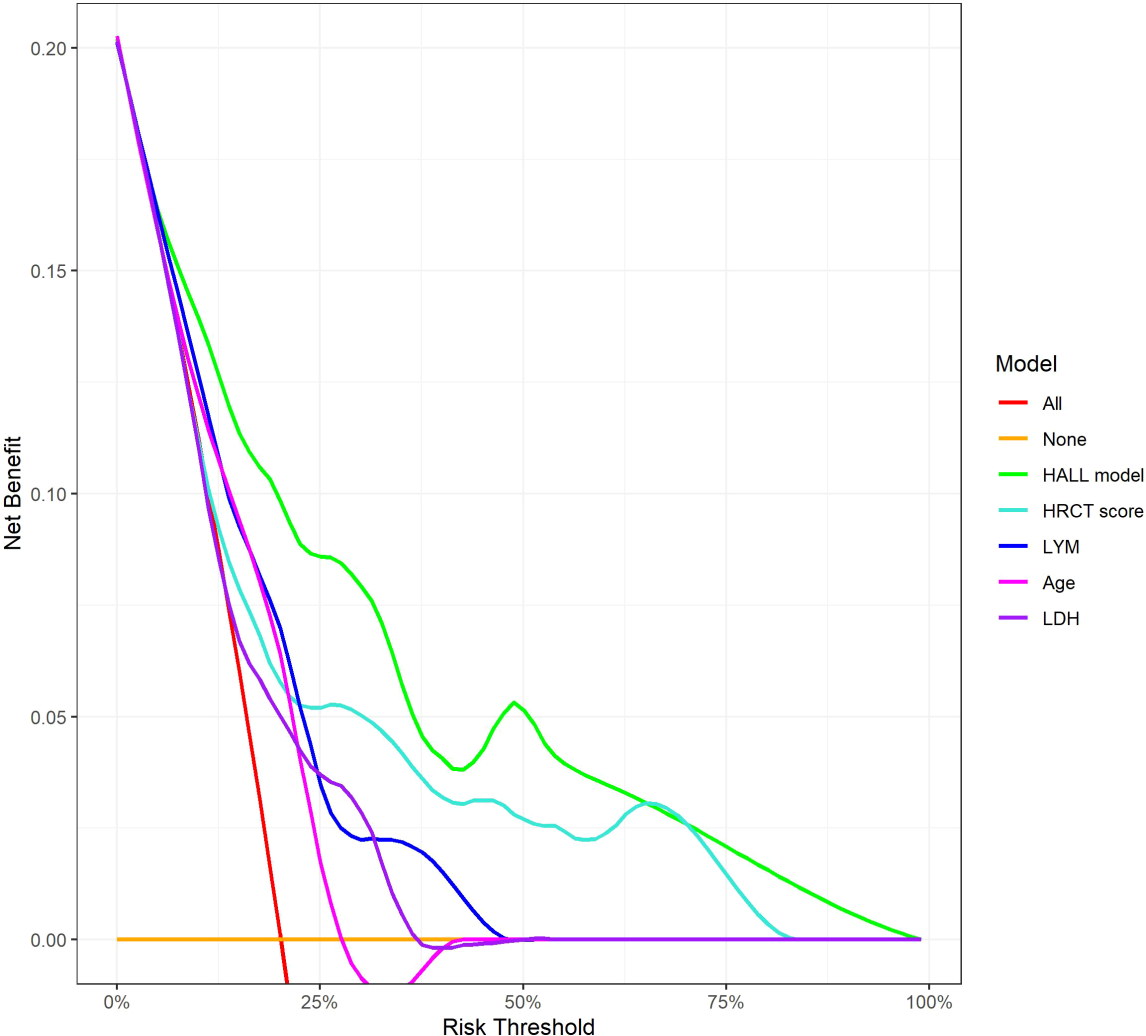
Decision Curve Analysis for 12-Month Infection Risk Prediction Models. This figure shows the net benefit of various predictive models across different risk thresholds. The lines represent different evaluated models: ‘HALL model’, compared with univariate predictors including HRCT score, LYM, Age, and LDH.

The AUC for predicting severe infections within six months was 0.84 (95% CI 0.76–0.92) in the derivation set and 0.91 (95% CI 0.79–1.02) in the validation set (**Figures 3A, B**). Additionally, the nomogram’s AUC for predicting infection risk at six months, one year, and three years were 0.84, 0.83, and 0.78, respectively, in the derivation set, and 0.91, 0.77, and 0.64, respectively, in the validation set (**Figures 3C, D**). We utilized the Cox proportional hazards model to calculate individual infection risk scores and evaluated the model’s predictive capability using ROC curves to determine the optimal cutoff value of 53.85. For practical purposes, this value was rounded to 54, and based on this cutoff, we stratified individuals into high-risk and low-risk groups. The Kaplan–Meier curves for these groups are shown in **Figures 3E** and **F**. The curves demonstrate good separation between high-risk and low-risk groups, indicating satisfactory discriminative ability. Furthermore, calibration curves for both the derivation and validation sets indicated a high degree of concordance between observed outcomes and those predicted by the nomogram (**Figure 4**). DCA revealed that the newly proposed model significantly increased the net benefit compared to univariate severe infection predictions. This analysis demonstrates that the nomogram provides a higher net benefit across a broader range of threshold probabilities, indicating its superior clinical utility in predicting severe infections in IIM patients (**Figure 5**).

### 3.5 Subgroup validation for DM and ASS

**Figure 6:**
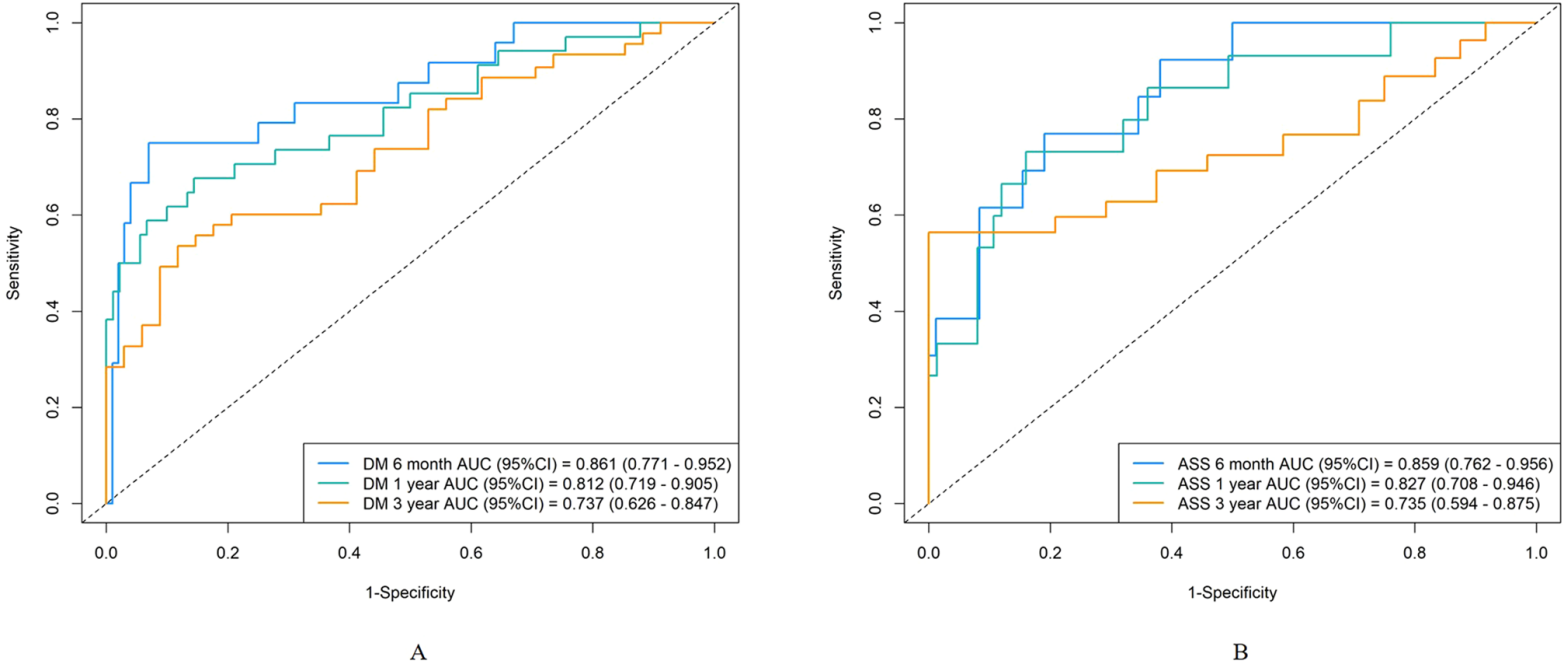
Discrimination of the nomogram in the DM and ASS subgroups. Receiver operating characteristic curves for 6-month, 1-year, and 3-year severe infection predictions in the DM subgroup **(A)** and ASS subgroup **(B)**.

**Figure 7:**
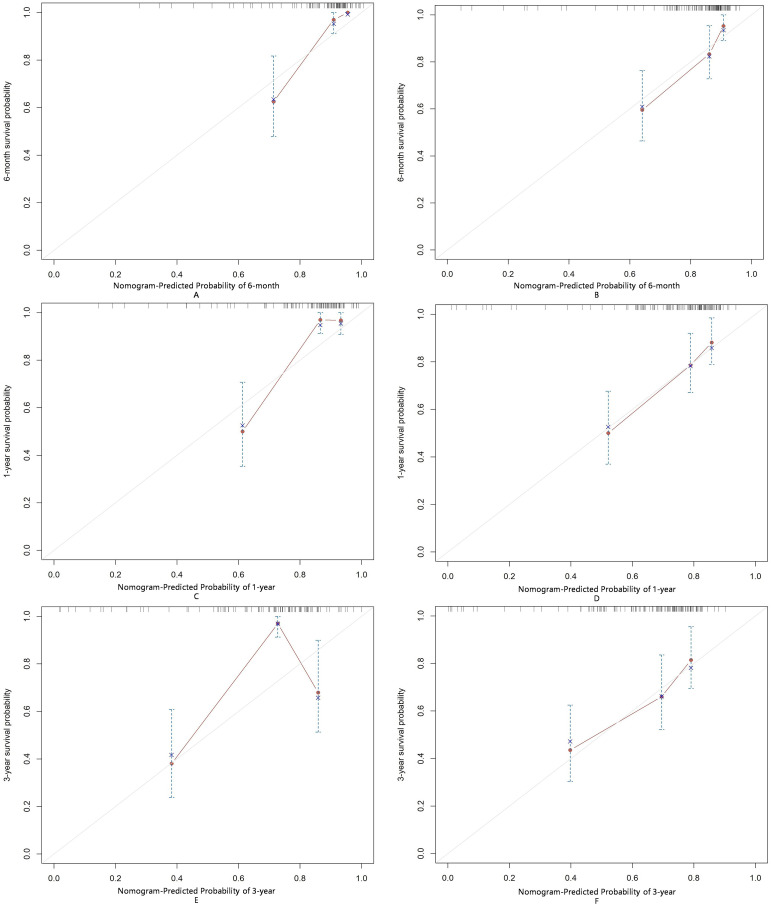
Calibration of the nomogram for infection predictions at 6-month **(A, B)**, 1-year **(C, D)**, and 3-year **(E, F)** intervals in the ASS and DM subgroups. Data are sourced from the ASS subgroup **(A, C, E)** and the DM subgroup **(B, D, F)**. The nomogram-predicted cumulative incidence rates of infection are stratified into equally sized subgroups. For each subgroup, the average predicted infection incidence rate is plotted against the observed infection incidence rate. Vertical lines represent the 95% confidence intervals for the infection incidence rates. The gray lines serve as reference lines, indicating the ideal position for the nomogram.

To further evaluate the applicability of the nomogram in different subtypes of IIMs, we performed subgroup analyses focusing on patients with DM and ASS. For the DM subgroup, the AUC for predicting severe infections within six months, one year, and three years were 0.86, 0.81, and 0.73, respectively (**Figure 6A**). For the ASS subgroup, the AUCs for the same time points were 0.86, 0.83, and 0.74, respectively (**Figure 6B**). Calibration curves for both DM and ASS subgroups indicated good concordance between observed outcomes and those predicted by the nomogram (**Figure 7**).

## 4 Discussion

In this cohort of 263 patients with IIMs, we explored the risk factors for severe infections and developed a nomogram to predict the 6-month, 1-year, and 3-year risks of severe infection. This nomogram incorporates a set of easily obtainable clinical risk factors, including age, LYM, LDH, and HRCT score for semi-quantitatively assessing ILD. Unlike previous studies that considered the presence of ILD as a risk factor for severe infection, our model provides a more refined prediction of severe infection risk. To our knowledge, this is the first risk prediction model developed and validated in an IIM patient cohort, and it could become a valuable tool in clinical practice.

In our cohort, 30.8% of IIM patients experienced infections, with respiratory infections being the most common. Bacterial infections remained the most frequent, consistent with findings from Chen et al. (14). In our cohort, viral infections, particularly CMV, had a high detection rate, aligning with previous studies indicating that CMV is the most common opportunistic infection pathogen (15). This may be due to the use of high-dose steroids and/or immunosuppressive drugs that facilitate CMV reactivation (16, 17). Zhao L et al. reported that DM patients with CMV infection had higher rates of ILD and mortality compared to those without CMV infection, highlighting the clinical importance of this issue (18). Pneumocystis jirovecii pneumonia (PJP) is another opportunistic fungal pathogen in immunosuppressed patients. A meta-analysis found that 6% of DM/PM patients developed PJP (19); however, the PJP infection rate in our cohort was 2.2%, possibly due to prophylactic use of low-dose trimethoprim-sulfamethoxazole in patients on high-dose steroids (20, 21), which warrants further investigation.

Patients who are positive for anti-MDA5 antibodies and those with ILD are considered high-risk for infections. Ge YP et al. suggested that IIM patients with anti-MDA5 antibodies (OR=1.93; 95% CI=1.20-3.11) and ILD (OR=2.03; 95% CI=1.30-3.71) are more prone to infections (9). In our univariate analysis, anti-MDA5 antibodies (HR=3.46; 95% CI=1.34-8.96) and ILD (HR=1.83; 95% CI=1.03-3.27) were significant predictors, but multivariate analysis did not show significance for anti-MDA5 antibodies (HR=1.90; 95% CI=0.64-5.60) and ILD (HR=0.92; 95% CI=0.37-2.29). This discrepancy may be due to the variability in ILD severity among different populations. Patients with anti-MDA5 antibodies may not always have severe ILD. You HX et al. categorized anti-MDA5 positive DM patients into three clinical subtypes based on their ILD risk (12). Our multivariate analysis did not show ILD as a significant factor, possibly due to differences in ILD severity, hence the use of HRCT scores to quantify ILD severity, which improves upon qualitative assessments. While our study did not find significant statistical differences in infection rates among IIM subtypes, univariate analysis indicated a protective tendency for PM (HR 0.28; 95% CI 0.07-1.16). This trend was less pronounced in DM, ASS, and IMNM, likely due to their association with severe ILD or significant muscle symptoms requiring high-dose steroids and intensive immunosuppression, which may increase infection risk (22, 23). High-dose glucocorticoid therapy has been identified as a risk factor for infections in IIM patients (9, 24, 25). In our study, univariate analysis showed a significant association between initial glucocorticoid dose and infection occurrence (HR=1.003; 95% CI=0.999-1.005), while multivariate analysis did not (HR=1.002; 95% CI=0.998-1.005). We hypothesize that adequate glucocorticoid therapy may improve lung function in IIM patients with ILD, facilitating rapid relief and reducing subsequent infection risk. This hypothesis requires further investigation.

Nomograms are commonly used in cancer prognosis as intuitive and user-friendly prediction tools. Previous studies have primarily used Cox regression to identify independent risk factors for severe infections without integrating these into a visual nomogram. In this study, we used the AIC-based subset Cox regression analysis to select the lowest AIC value risk factors (lymphocyte count, HRCT score, lactate dehydrogenase, and age) to develop a nomogram predicting severe infection in IIM patients. The nomogram’s validity was assessed through discrimination and calibration. The AUC for predicting 6-month and 1-year severe infection risk exceeded 0.7, while the 3-year AUC was 0.639, likely due to the limited sample size and low cumulative event rate at 3 years. Calibration curves demonstrated good prediction accuracy for 6-month and 1-year severe infection risks in both the DM and ASS subgroups. However, at 3 years, the calibration curves indicated suboptimal prediction accuracy, with a more pronounced deviation from the ideal line observed in the ASS subgroup compared to the DM subgroup. This suggests that the nomogram’s long-term predictive performance may be less reliable in patients with ASS. DCA analysis revealed that, compared to single-variable infection predictions, the newly proposed model significantly increased net benefit and exhibited a wider range of threshold probabilities. This indicates that the nomogram not only accurately predicts the risk of severe infections but also provides greater clinical value by aiding in decision-making processes. By considering a range of threshold probabilities, the model helps clinicians balance the benefits of early intervention against the risks of overtreatment, thereby optimizing patient management.

Despite the model’s good performance in the derivation and validation sets, its limitations are noteworthy. All participants were from a single center, limiting external validity. Subgroup analyses revealed that the 3-year prediction accuracy was lower compared to the 6-month and 1-year predictions, as indicated by the calibration curves. The ASS subgroup, in particular, exhibited a more pronounced deviation from the ideal line, reflecting poorer long-term predictive performance. This may be due to the smaller sample size and lower cumulative event rates in the ASS subgroup, as well as the clinical heterogeneity of anti-synthetase syndrome, including variations in disease severity and treatment regimens over time. Internal validation may lead to overfitting, restricting model performance on new datasets. The small sample size (263 cases) might result in statistical instability and bias. Therefore, larger multicenter studies are needed for external validation to assess the model’s robustness and generalizability. Additionally, employing dynamic modeling techniques that account for time-dependent variables could improve the nomogram’s long-term predictive performance, particularly in clinically heterogeneous subgroups such as patients with ASS. While the model shows promising performance, the absence of prospective cohort validation highlights an area for further improvement. Prospective studies could confirm its predictive reliability and robustness in real-world settings, enhancing its clinical applicability. Additionally, as treatment evolves over time, the overall event rate may change, potentially causing the model to become obsolete. Regular updates and performance reassessments are necessary to ensure its ongoing clinical relevance. While the model shows promise, there is room for improvement. Future studies should include data from other centers to evaluate the model’s applicability across different external validation cohorts. Moreover, our study relied on traditional pathogen detection methods, lacking next-generation sequencing, which may lead to inaccuracies in infection diagnosis and classification. Introducing new pathogen detection techniques could enhance detection rates, facilitate early identification of infections, and aid in developing more effective clinical strategies.

## 5 Conclusion

We have developed a nomogram to predict the risk of severe infections in patients with IIMs. This nomogram includes four clinical variables: age, LYM, LDH, and HRCT score. It provides clinicians with a simple and practical tool to assess the risk of severe infections in IIM patients over 6 months, 1 year, and 3 years, facilitating early identification of high-risk patients and aiding in the formulation of appropriate preventive and therapeutic strategies.

## Data Availability

The original contributions presented in the study are included in the article/supplementary material. Further inquiries can be directed to the corresponding authors.
